# Molecular Characteristics, Clinical Significance, and Cancer Immune Interactions of Angiogenesis-Associated Genes in Gastric Cancer

**DOI:** 10.3389/fimmu.2022.843077

**Published:** 2022-02-22

**Authors:** Xin Qing, Wenjing Xu, Shengli Liu, Zhencheng Chen, Chunping Ye, Yewei Zhang

**Affiliations:** ^1^ School of Medicine, Zhongda Hospital, Southeast University, Nanjing, China; ^2^ Hepatopancreatobiliary Center, The Second Affiliated Hospital of Nanjing Medical University, Nanjing, China; ^3^ School of Life and Environmental Sciences, Guilin University of Electronic Technology, Guilin, China; ^4^ Department of Obstetrics and Gynecology, Nanjing Maternity and Child Health Care Hospital, Women’s Hospital of Nanjing Medical University, Nanjing, China

**Keywords:** gastric cancer, angiogenesis, prognosis, tumor microenvironment, immunotherapy

## Abstract

**Background:**

Immunotherapy has evolved as a critical option to treat diverse cancers. The active response to immunotherapy relies on the unique interaction between cancer and the tumor microenvironment (TME). Angiogenesis is one of the hallmarks of cancer. However, the association between angiogenesis and clinical outcome, immune cell infiltration, and immunotherapy remains unknown in gastric cancer (GC).

**Methods:**

We systematically assessed 36 angiogenesis-associated genes (AAGs) and comprehensively identified the correlation between angiogenesis and transcriptional patterns, prognosis, and immune cell infiltration. The AAG_score was applied to quantify the angiogenesis subtypes of each patient. We then evaluated their values in prognostic prediction and therapeutic responses in GC.

**Results:**

We discussed the mutations of AAGs in GC specimens from genetic levels and identified their expression patterns from TCGA and GEO cohorts. We determined two different molecular subtypes and observed that AAG mutations were related to patients’ clinicopathological characteristics, prognosis, and infiltrating TME. Next, an AAG_score for predicting overall survival (OS) was established and its reliable predictive ability in GC patients was confirmed. Furthermore, we created a highly reliable nomogram to facilitate the clinical viability of the AAG_score. A low AAG_score, characterized by elevated microsatellite instability-high, mutation burden, and immune activation, demonstrated a superior OS. Additionally, the AAG_score was remarkedly correlated with the cancer stem cell index and drug susceptibility.

**Conclusion:**

Collectively, we identified a prognostic AAG signature for GC patients. This signature may contribute to clarifying the characteristics of TME and enable the exploration of more potent immunotherapy strategies.

## Introduction

Immunotherapy is a blooming treatment modality for diverse tumors, and its effectiveness against tumors is being confirmed by a growing body of clinical studies (1–3). Common immunotherapeutic strategies include ICP inhibitors (ICIs), therapeutic antibodies, and cell therapy. The studies of ICIs for PD-1, PD-L1, and CTLA-4 are emerging and clinical reports have proven their safety and effectiveness (4, 5). However, persistent benefits were only realized in a minority of patients. Accumulative studies demonstrate that the tumor microenvironment (TME) is responsible for the aggressive behaviors of tumors and affects the tumor response for immunotherapy (6). The TME consists of various factors, including tumor cells, blood vessels, infiltrating immune cells, stromal cells, tissue fluid, and cytokines (7). The formation of new blood vessels is a hallmark of TME and is characterized by continuous and disordered. Typically, tumor cells promote angiogenesis and inflammation, thus evading the surveillance and clearance of the immune system (8). Therefore, global analysis of the relationship between angiogenesis and TME can discover different neoplastic immunophenotypes and boost the predictive power of immunotherapy.

Gastric cancer (GC), a prevalent malignancy, has a rapid increase in incidence annually (9). Despite advances in chemotherapeutic regimens for advanced GC, such as 5-FU-based regimen and platinum-based regimen, chemotherapy effects remain unsatisfactory, with overall survival (OS) struggling to exceed 2 years (10, 11). Accordingly, targeted therapy is a future development direction to target GC. In recent years, various targeted drugs have been developed, however, overall results remain disappointing (12). Immunotherapy offers additional options for GC patients and brings hope for the treatment of GC. Although immunotherapy has brought huge benefits to GC patients, it has also been found that specific types of patients benefit from immunotherapy (13). It is necessary to develop valuable biomarkers that can classify patients with different characteristics into diverse groups and predict the effect of immunotherapy.

Angiogenesis is one of the crucial elements to support tumor growth and development, and various angiogenic factors tend to be overexpressed (14). Recently, the inhibition of angiogenesis has emerged as an encouraging therapeutic option, particularly for tumors where conventional treatment is unavailable (15). However, the majority of the present studies are focused on identifying the role of individual angiogenesis-associated genes (AAGs) on the progression and prognosis of GC. In addition, Expression proteins of AAGs are often used as therapeutic targets for tumors, and exploring the relationship between AAGs and tumor innate immune may contribute to further combining targeted therapy and immunotherapy (16, 17).

We systematically analyzed the expression of AAGs and their impact on the development, prognosis, TME, and therapeutic response of GC patients. We identified three distinct angiogenesis subgroups in GC with the TCGA database and GEO database. Next, we assessed the molecular characteristics, prognostic significance, and infiltrating immune cell intensities of the identifying angiogenesis clusters. Furthermore, we obtained an AAG_score that accurately predicted the clinical outcome of GC patients and immunotherapeutic effect. We expect that this study will contribute to the development of viable immunotherapies for GC.

## Materials and Methods

### Data Collection

The RNA expression data, somatic mutation data, CNV files, and corresponding clinicopathological information of GC were retrieved from the TCGA-STAD program, and GSE84337 from the GEO repository was utilized to acquire clinical parameters and normalized gene expression data (18). Samples lacking significant clinicopathological or survival information were excluded from further analysis. 36 AAGs were obtained from the MSigDB Team (Hallmark Gene set) (**Table S1**).

### Consensus Clustering Analysis of AAGs

Consensus clustering was employed to define distinct angiogenesis-related patterns by the k-means algorithms (19). The quantity, as well as consistency of clusters, were built by the consensus clustering algorithm, which is available in the “ConsensuClusterPlus” package (20). 1000 iterations were performed to ensure the stability of these categories. To identify the biological functional differences in AAGs, gene set variation analysis (GSVA) was conducted with the KEGG gene set (c2.cp.kegg.v7.4) (21).

### Association Between Molecular Patterns With the Clinical Characteristics and Prognosis of GC

To determine the clinical significance of the clusters generated by consensus clustering, we investigated the association between molecular patterns, clinical features, and survival outcomes. The clinical variables included age, gender, T-stage, and N-stage. Moreover, the differences in OS between different patterns were evaluated with Kaplan–Meier analysis obtained by the “survival” and “survminer” packages (22).

### Relationship of Molecular Patterns With TME in GC

We assess the immune and stromal scores of GC patients with the ESTIMATE algorithm (23). Next, the levels of 22 immune cell subtypes of each patient were computed with the CIBERSORT algorithm (24). The infiltrating fractions of immune cells were also identified with a single-sample gene set enrichment analysis (ssGSEA) algorithm (25). We then evaluated the association between the two subsets on PD-1, PD-L1, and CTLA-4 expression.

### Identification of DEGs and Functional Enrichment Analysis

To identify DEGs in the distinct angiogenesis subgroups, we used the “limma” package with criteria of |log2-fold change (FC)| ≥ 1 and p-value < 0.05. On the basis of these DEGs, GO and KEGG analysis was carried out with the “clusterProfiler” package (26).

### Development of the Angiogenesis-Associated Prognostic AAG_Score

An AAG_score was constructed to quantitatively assess angiogenesis in individual GC patients. The expression data of DEGs from distinct angiogenesis clusters were standardized across GC specimens and the intersect genes were selected. The differential assessment demonstrated that there are 234 DEGs between the two angiogenesis clusters. Next, we conducted univariate Cox regression (uniCox) analysis for DEGs. Survival-related genes were retained for further analysis. We carried on principal component analysis (PCA) to generate angiogenesis-associated gene scores with the following algorithm: AAG_score = expression of a gene [1] × corresponding coefficient [1] + expression of a gene [2] × corresponding coefficient [2] + expression of gene [n] × corresponding coefficient [n].

### Clinical Significance and Classification Analysis of the Prognostic AAG_Score

The relevance of the AAG_score to clinical variables was investigated. To identify whether AAG_score was an independent prognostic predictor, we conducted uniCox and multivariate Cox regression (multiCox) analysis for all cohorts. Then, we conducted a classification analysis to explore whether the AAG_score remains its predictive reliable in distinct subgroups based on multiple clinical variables. Furthermore, the infiltrating levels of immune cells and immune checkpoint (ICP) were compared in the different risk score subgroups. Additionally, we examined the correlations between AAG_score and tumor mutation burden (TMB) score, microsatellite instability (MSI) score, and cancer stem cells (CSC) score.

### Establishment of a Predictive Nomogram

A nomogram was depicted to provide valuable clinical predictions for HCC patients with their risk scores and other clinicopathological characteristics, particularly about 1-, 3-, and 5-year OS. Next, we performed calibration curve analysis and decision curve analysis (DCA) to verify the clinical reliability of the established nomogram.

### Mutation and Drug Sensitivity Analysis

To identify the mutational profiles of GC patients between different risk groups, the mutation annotation format (MAF) from the TCGA database was created with the “maftools” package (27). We also assessed tumor immune dysfunction and exclusion (TIDE) and immunophenotype score (IPS) for GC patients in the two groups. To investigate the clinic performance of chemotherapy agents in patients, we computed the semi-inhibitory concentration (IC50) values of common drugs with the “pRRophetic” package (28).

### Statistical Analysis

R software (version 4.1.2) and its relevant packages are applied to process, analyze and present the data. A two-sided P <0.05 was deemed valuable.

## Results

### Genetic Mutation Landscape of AAGs in GC

**Figure 1:**
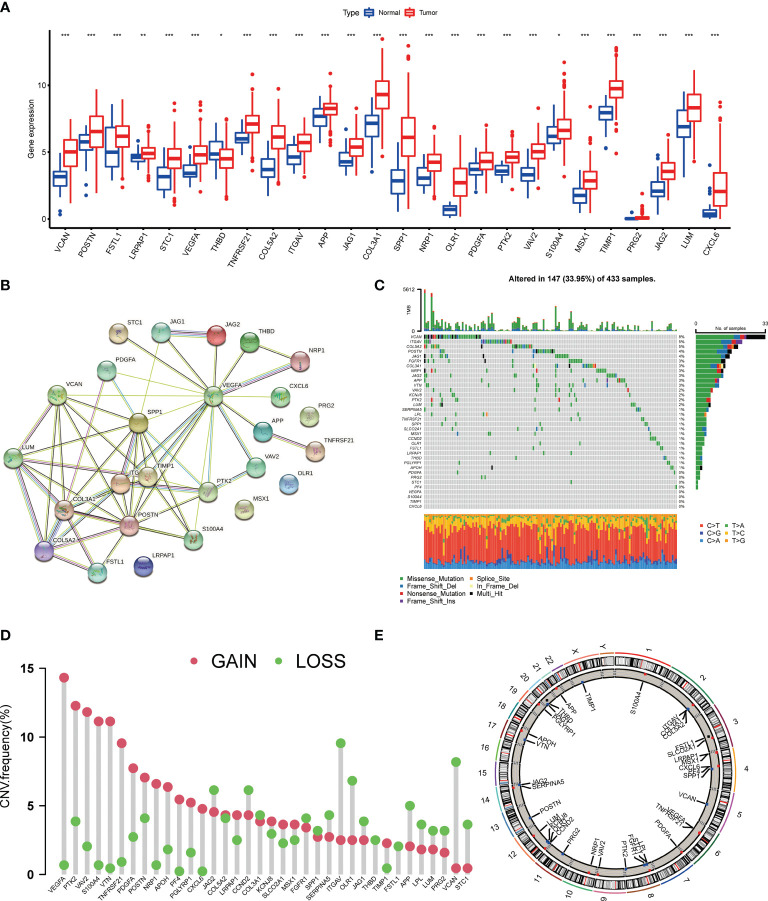
Genetic mutational landscape of AAGs in GC. **(A)** Expression distributions of DEGs between GC and normal tissues. **(B)** The PPI network acquired from the STRING database among the DEGs. **(C)** Genetic alteration on a query of AAGs. **(D)** Frequencies of CNV gain, loss, and non-CNV among AAGs. **(E)** Circus plots of chromosome distributions of AAGs. (p < 0.05 *; p < 0.01 **; p < 0.001 ***).

We first identified the expression levels of the 36 AAGs in tumor specimens and normal specimens with the TCGA-STAD dataset. A total of 26 DEGs were found, and most of the DEGs were abundant in the tumor samples (**Figure 1A**). A protein-protein interaction (PPI) analysis through the string website was established to reveal the interactivity of DEGs, which indicated that VEGFA, SPP1, POSTN, VTN, COL3A1, and TIMP1 were hub genes (**Figure 1B**). Next, we determined the incidence of CNVs and somatic mutations of 36 AAGs in GC. As depicted in **Figure 1C**, 147 of 433 (33.95%) GC samples presented genetic mutations, and the findings suggested VCAN as the gene with the highest mutation incidence, followed by ITGAV and COL5A2, among the 36 AAGs. Furthermore, we explore CNV mutational incidence, which indicated that 36 AAGs demonstrated evident CNV alterations (**Figure 1D**). **Figure 1E** displays the site of CNV alterations of 36 AAGs on chromosomes. We summarized that CNV may serve a regulative role in the expression of AAGs. The findings indicated a substantial difference in the genomic background and expression levels of AAGs between GC and normal specimens, suggesting the potential role of AAGs in GC tumorigenesis.

### Generation of Angiogenesis Subgroups in GC

**Figure 2:**
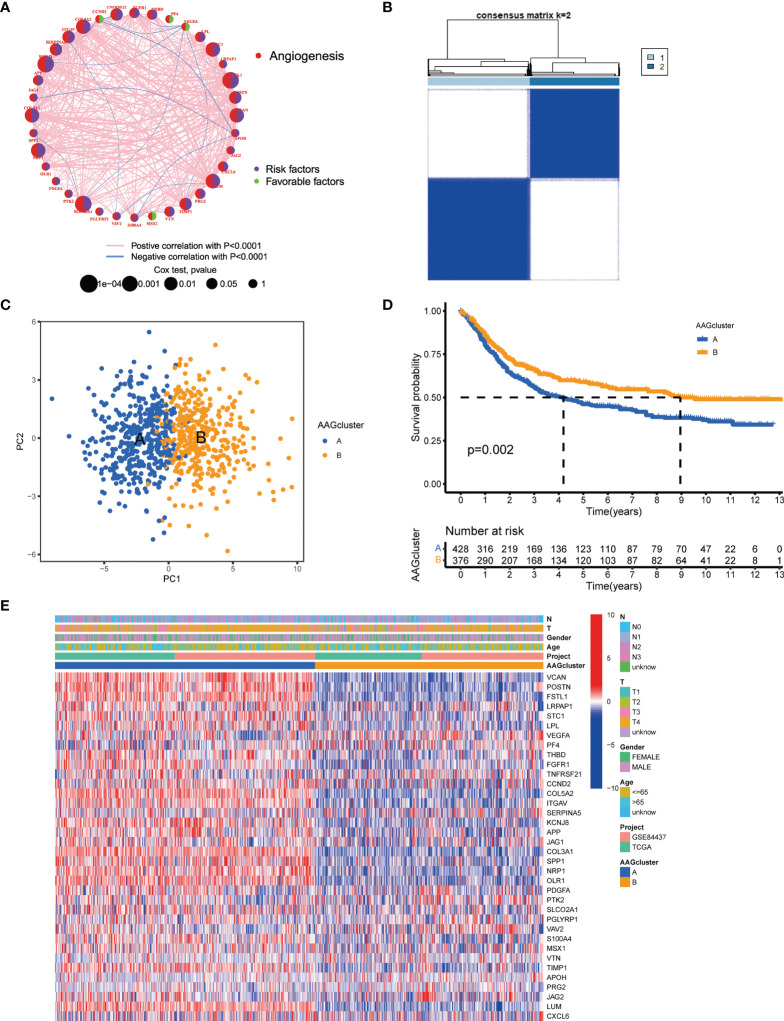
AAG subgroups and clinicopathological and biological characteristics of two distinct subtypes of samples divided by consistent clustering. **(A)** A network of correlations including AAGs in the TCGA cohort. **(B)** Consensus matrix heatmap defining two clusters (k = 2) and their correlation area. **(C)** PCA analysis indicating an obvious difference in transcriptomes between the two subgroups. **(D)** Univariate analysis showing 36 AAGs correlated with OS. **(E)** Differences in clinicopathologic characteristics and expression levels of AAGs between the two distinct subgroups.

The detailed flowchart of this work is shown in **Figure S1**. 804 GC patients from TCGA-STAD and GSE84437 were enrolled in this study to reveal the relationship between angiogenesis and tumorigenesis. Complete information of these patients was listed in **Table S2**. The prognostic values of 36 AAGs in GC patients were identified with uniCox and Kaplan–Meier analysis (**Table S3**). Next, the correlation network of AAG interactions, regulator relationships, and their survival significance in GC patients was presented in **Figure 2A**, and **Table S4**.

To further determine the relationship between expression patterns of AAGs and GC subtypes, we performed a consensus clustering analysis to classify GC patients according to the expression levels of these AAGs. Our findings indicated that the optimal clustering variable was 2 (**Figure 2B**), and GC patients in the entire cohort were well dispersed in cluster A (n=430) and cluster B (n=378). The result of PCA analysis also confirmed the excellent intergroup distribution (**Figure 2C**). Furthermore, the OS time of the two clusters was discussed, and a significant survival difference was observed (**Figure 2D**). Additionally, as displayed in **Figure 2E**, the genomic expression and clinicopathological variables of both clusters were compared, and a substantial difference of AAGs expression and clinical features were identified.

### Characteristics of the TME in Different Subgroups

**Figure 3:**
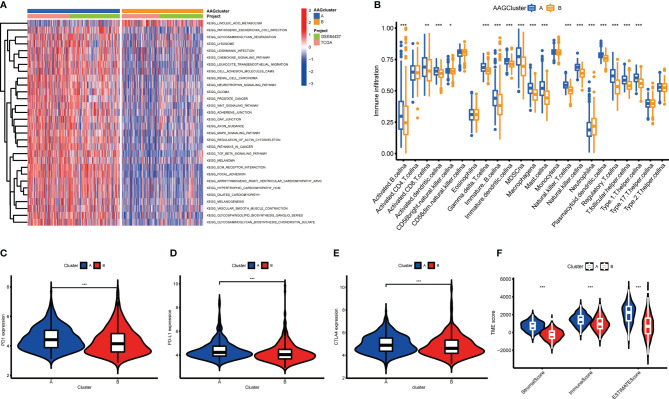
Correlations of tumor immune microenvironments and two GC subgroups. **(A)** GSVA of biological pathways between two distinct subgroups. **(B)** Abundance of 23 infiltrating immune cell types in the two GC subgroups. **(C–E)** Expression levels of PD-1, PD-L1, and CTLA-4 in the two GC subgroups. **(F)** Correlations between the two GC subgroups and TME score. (p < 0.05 *; p < 0.01 **; p < 0.001 ***).

According to the findings of GSVA analysis, cluster A was abundant in cancer-associated pathways (multiple cancer such as renal cell carcinoma, glioma, prostate cancer, and melanoma) and metastasis-associated pathways (regulation of cell adhesion molecules cams, ECM receptor interaction, and focal adhesion) (**Figure 3A** and **Table S5**). To identify the relationship between AAGs and the TME of GC, we explore the infiltrating levels of 23 human immune cell subpopulations in the two clusters with the CIBERSORT algorithm (**Table S6**). As shown in **Figure 3B**, a substantial enrichment difference of most immune cells between both clusters was noticed. The enrichment levels of activated B cell, activated CD8 T cell, activated DC cell, CD56bright NK cell, gd T cell, immature B cell, immature DC cell, MDSC, macrophage, mast cell, NK T cell, NK cell, plasmacytoid DC cell, regulatory T cell, T follicular helper cell, and type 1 T helper cell were markedly higher in the cluster A than cluster B, while the opposite performance of neutrophil was observed. Moreover, the expression of three critical ICPs (PD-1, PD-L1, and CTLA-4) was notably greater of cluster A than cluster B (**Figures 3C–E**). And TME scores could evaluate the abundance of immune and stromal elements in TME, we further executed the ESTIMATE algorithm to obtain the TME scores in the different clusters, including stromal score, immune score, and estimate score. The findings indicated patients in cluster A had higher TME scores (**Figure 3F**).

### Identification of Gene Subgroups Based on DEGs

**Figure 4:**
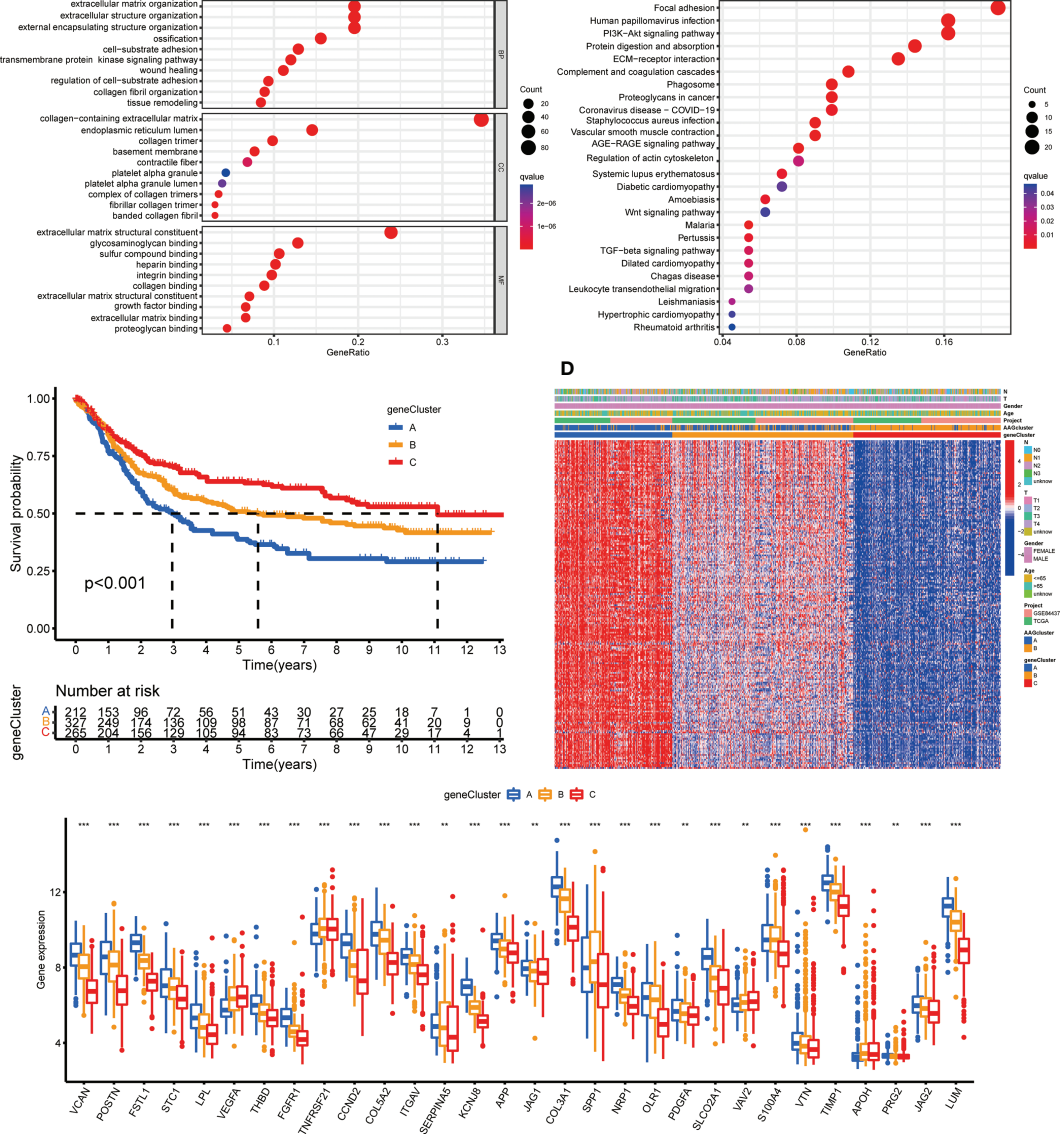
Identification of gene subgroups based on DEGs. **(A, B)** GO and KEGG enrichment analyses of DEGs among two angiogenesis subgroups. **(C)** Kaplan–Meier curves for OS of the three gene clusters. **(D)** Relationships between clinicopathologic features and the three gene clusters. **(E)** Differences in the expression of 36 AAGs among the three gene clusters. (p < 0.01 **; p < 0.001 ***).

To investigate the underlying biological activity of angiogenesis subgroups, we obtained 234 angiogenesis clusters-associated DEGs with the “limma” package and conducted functional enrichment analysis (**Table S7**). These angiogenesis subgroups-associated DEGs were mainly enriched in metastasis-associated biological processes (**Figure 4A**). KEGG analysis demonstrated the abundance of cancer- and metastasis-associated pathways (**Figure 4B**), implying that angiogenesis serves as a crucial factor in the modulation of tumor metastasis. Then, we performed uniCox analysis to determine the survival significance of these genes, and 204 genes were extracted with a criterion of p < 0.05 (**Table S8**). To investigate specific adjustment mechanisms, a consensus clustering method was utilized to separate patients into different gene clusters (Clusters A-C) on the basis of prognostic genes (**Figure S2**). Kaplan-Meier analysis demonstrated that patients in cluster A had the shortest OS time, whereas patients in cluster C had the superior OS time (**Figure 4C**). Additionally, angiogenesis gene cluster A patterns were related to advanced T- and N-stage (**Figure 4D**). The angiogenesis gene clusters demonstrated substantial discrepancies in AAGs expression, as expected from the angiogenesis subgroups (**Figure 4E**).

### Development and Validation of the Prognostic AAG_Score

**Figure 5:**
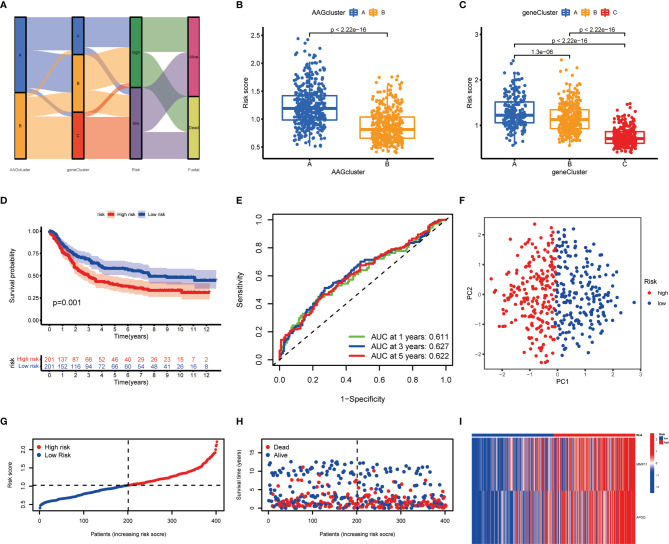
Construction of the AAG_score in the training cohort. **(A)** Alluvial diagram of subgroup distributions in groups with different AAG_scores and clinical outcomes. **(B)** Differences in AAG_score between the two angiogenesis clusters. **(C)** Differences in AAG_score between the three gene clusters. **(D)** Kaplan–Meier analysis of the OS between the two groups. **(E)** ROC curves to predict the sensitivity and specificity of 1-, 3-, and 5-year survival according to the AAG_score. **(F)** PCA analysis based on the prognostic signature. **(G, H)** Ranked dot and scatter plots showing the AAG_score distribution and patient survival status. **(I)** Expression patterns of 2 selected prognostic genes in high- and low-risk groups.

The AAG_score was created on the basis of cluster-associated DEGs. The GC patients were randomly assigned into a training cohort (n=402) or a test cohort (n=402) at a ratio of 1:1. LASSO and multivariate Cox (multiCox) analysis for 204 angiogenesis cluster-associated prognostic DEGs were conducted to establish an optimal predictive model (**Figure S3**). Ultimately, we acquired two genes (MMP11 and APOD), and the AAG_score was accessed as described: Risk score = (0.1347* expression of MMP11) + (0.1099* expression of APOD). **Figure 5A** displayed the patients’ distribution in the two angiogenesis clusters, three gene clusters, and two AAG_score groups.

We discovered a substantial difference in the AAG_score of the angiogenesis clusters and gene clusters (**Figures 5B, C**). We observed the highest AAG_score in gene cluster A and the lowest AAG_score in gene cluster C, implying a low AAG_score may be correlated with immune activation-associated characteristics. Based on the abovementioned survival analysis, we identified that higher risk scores of both classifications were correlated with worse survival. Furthermore, Kaplan-Meier analysis in the training cohort indicated that low-risk patients had a better OS over high-risk patients (**Figure 5D**), and the AUCs of 1-, 3-, and 5-years OS were 0.611, 0.627, and 0.622, respectively (**Figure 5E**). PCA analysis revealed a clear distribution between the two risk groups (**Figure 5F**). The risk plot of AAG_score indicated that as AAG_score increased, OS time decreased while mortality rise (**Figures 5G, H**). Additionally, a heatmap of selected genes was presented in **Figure 5I**.

To evaluate the predictive robustness of AAG_score, we obtained AAG_score of the test cohort and entire cohort (**Figures S4**, **S5**). The patients were also assigned into different risk subgroups depending on the median score of the training cohort. Similarly, survival analysis presented a superior OS of low-risk patients compared to high-risk patients. Prediction of the 1-, 3-, and 5-year survival probability suggested that the AAG_score still had excellent AUC values, implying that the AAG_score had a great performance to assess the prognosis of GC patients.

### Clinical Correlation Analysis of the Prognostic AAG_Score

To determine the relationship of the AAG_score with clinicopathological features, we discussed the interaction between AAG_score and diverse clinical parameters (age, gender, T-stage, N-stage, and survival status). We found increased risk scores in the higher T- and N-stage (**Figure S6**). Furthermore, the independent prognostic value of AAG_score for GC patients was evaluated. We performed uniCox and multiCox analyses to explore prognostic independence of multiple clinical factors. As presented in **Figure S7**, age, T-stage, N-stage, and risk score in the training cohort demonstrated significant differences, which were concordant with the findings available in the test cohort and entire cohort (**Figure S7**). Moreover, to further explore the prognostic significance of AAG_scores in GC patients, the patients were assigned into different subgroups based on clinical parameters. Overall, the high-risk patient’s survival was generally poorer compared to low-risk patients (**Figure S8**).

### Construction of a Nomogram to Predict Patients’ Prognosis

**Figure 6:**
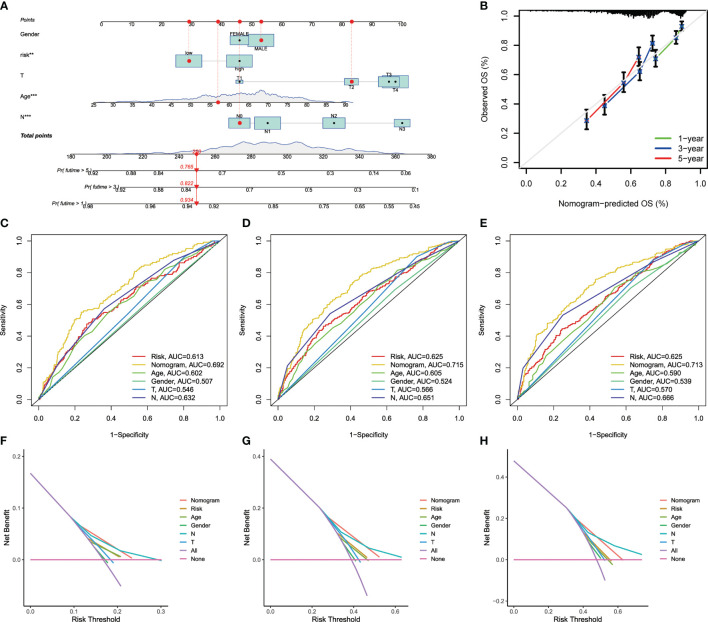
Construction and validation of a nomogram. **(A)** Nomogram for predicting the 1-, 3-, and 5-year OS of GC patients in the entire cohort. **(B)** ROC curves for predicting the 1-, 3-, and 5-year ROC curves in the entire cohort. **(C–E)** The time−dependent ROC curves of the nomograms compared for 1−, 3−, and 5−year OS in GC, respectively. **(F–H)** The DCA curves of the nomograms compared for 1−, 3−, and 5−year OS in HCC, respective.

Due to the high correlation between risk scores and patients’ prognosis, we incorporated clinical parameters to establish a nomogram. This nomogram was utilized to estimate 1-, 3-, and 5-year OS for GC patients (**Figure 6A**). The calibration curves of this established nomogram presented great accuracy between actual observations and predicted values (**Figure 6B**). Furthermore, we estimated the AUC values of these clinical factors for predicting OS at 1-, 3-, and 5-year, respectively. As shown in **Figures 6C–E**, the AUC values were as expected, implying this nomogram had an excellent predictive ability for prognosis. Moreover, we also found that this prognostic model with diverse clinical factors presented more net benefits for predicting the prognosis (**Figures 6F–H**). Additionally, we also compared AAG_scores and previously reported prognostic prediction models (29, 30), and the results showed AAG_scores had a superior predictive performance (**Figure S9**).

### Assessment of TME and Checkpoints in Distinct Groups

**Figure 7:**
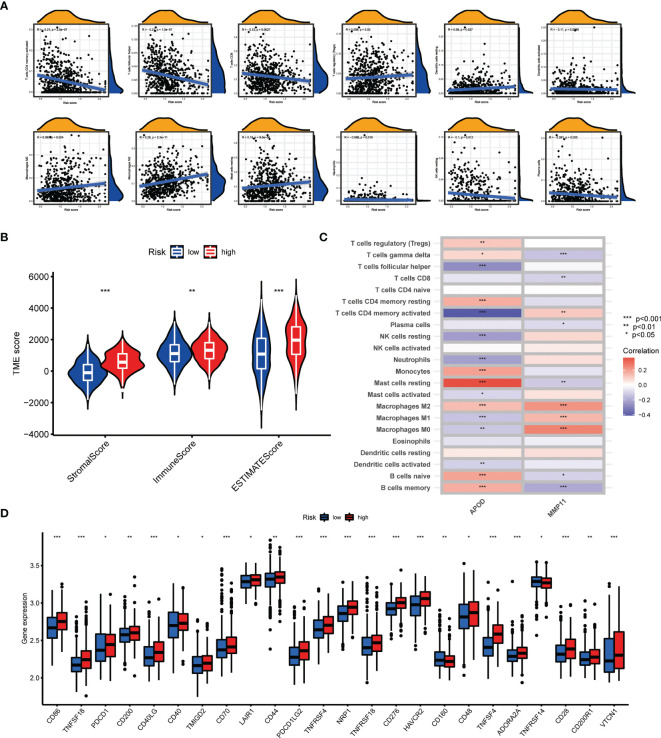
Evaluation of the TME and checkpoints between the two groups. **(A)** Correlations between AAG_score and immune cell types. **(B)** Correlations between AAG_score and both immune and stromal scores. **(C)** Correlations between the abundance of immune cells and selected genes in the prognostic model. **(D)** Expression of immune checkpoints in the high and low-risk groups. (p < 0.05 *; p < 0.01 **; p < 0.001 ***).

The CIBERSORT algorithm was utilized to evaluate the correlation between AAG_score and immune cells abundance. As depicted in **Figure 7A**, the AAG_score was positively associated with the infiltration of regulatory T cells, resting mast cells, M0 macrophages, M2 macrophages, and resting dendritic cells, while the opposite performance was observed in relationship with AAG_score and follicular helper T cells, CD8 + T cells, activated memory CD4 + T cells, plasma cells, resting NK cells, neutrophils, and activated dendritic cells. Moreover, the AAG_score was positively linked to stromal score, and immune score (**Figure 7B**). We then explore the correlation between the selected genes in the prognostic signature and the enrichments of immune cells. We concluded that the majority of immune cells were closely related to the selected genes (**Figure 7C**). Additionally, we assessed the relationship between ICPs and this prognostic signature. **Figure 7D** demonstrates that 24 ICPs were discrepantly represented in the two risk subgroups, such as PD-1, LAIR1, and VTCN1.

### Association of AAG_Score With TMB, MSI, and CSC Score

**Figure 8:**
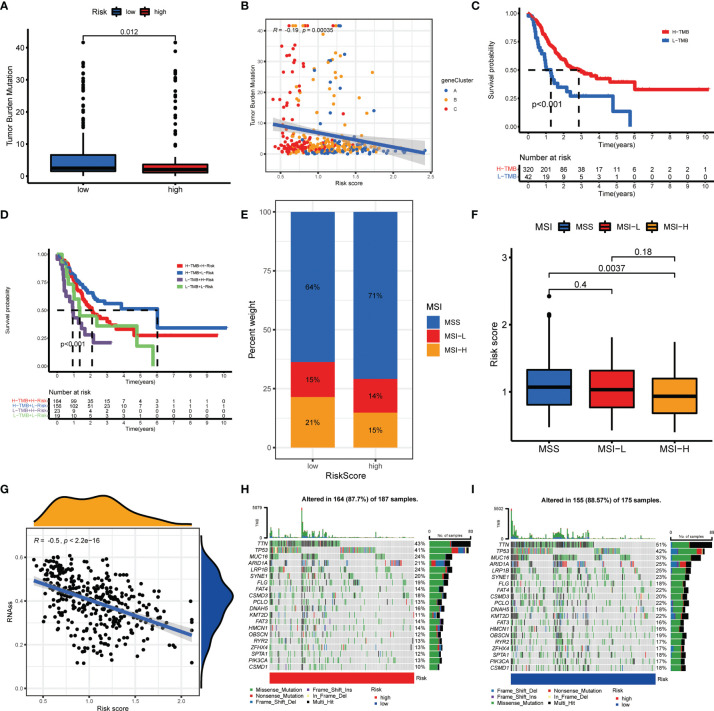
Comprehensive analysis of the AAG_score in GC. **(A, B)** Relationships between AAG_score and TMB. **(C)** Kaplan–Meier analysis of the OS between the low- and high-TMB groups. **(D)** Survival analysis among four patient groups stratified by both TMB and AAG_score. **(E, F)** Relationships between AAG_score and MSI. **(G)** Relationships between AAG_score and CSC index. **(H, I)** The waterfall plot of somatic mutation features established with high and low AAG_scores.

Numerous studies revealed that TMB and MSI were valuable predictive indicators for tumor immune response, and patients with high TMB or high MSI can benefit from ICP inhibitors (31–33). Our findings demonstrated a higher TMB in the low-risk groups over high-risk groups (**Figure 8A**), suggesting that low-risk patients may benefit more from immunotherapy. A negative correlation of AAG_score and TMB was also observed with Spearman correlation analysis (**Figure 8B**). To explore the impact of TMB status on prognosis in GC patients, we also conducted survival analysis across different TMB subgroups. High-TMB patients had a superior prognosis than low-TMB patients (**Figure 8C**). Subsequently, we combined TMB and AAG_score for survival analysis of GC patients, and the prognostic benefit in the high-TMB group was eliminated by the AAG_score (**Figure 8D**). Similarly, correlation evaluation demonstrated that a low AAG_score was linked to MSI-H pattern, while a high AAG_score was related to the microsatellite stable (MSS) pattern (**Figures 8E, F**). These results also suggested that low-risk patients may be more sensitive to immunotherapy. Furthermore, we integrated the AAG_score and CSC score to evaluate their latent relevance in GC. The relationship between AAG_score and CSC score was presented in **Figure 8G**. We summarized that AAG_score was negatively related to the CSC score, suggesting that GC cells with lower AAG_score had more prominent stem cell characteristics and a lower level of cell differentiation. Additionally, we investigated the distribution differences of the somatic mutations between AAG_score patterns in the TCGA-STAD dataset. As presented in **Figures 8H, I**, the mutation incidences of TP53, TTN, MUC16, ARID1A, LRP1B, and SYNE1 were higher than or equal to 20% in GC patients in two risk groups. Interestingly, these genes were mutated at a greater possibility in the low-risk group versus the high-risk group.

### Drug Sensitivity Analysis

**Figure 9:**
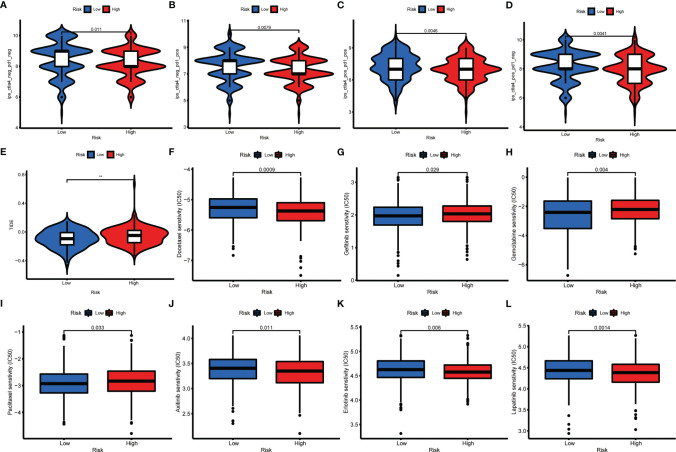
Relationships between AAG_score and therapeutic sensitivity. **(A–D)** IPS in different AAG_score groups. **(E)** TIDE in different AAG_score groups. **(F–L)** Relationships between AAG_score and chemotherapeutic sensitivity. (p < 0.01 **).

For unresectable GC patients, chemotherapy, targeted therapy, and immunotherapy may limit tumor progression and improve patients’ prognoses (34). To assess the immune response of GC patients, we calculated TIDE scores and IPS scores to predict patients’ response-ability. As shown in **Figures 9A–E**, low-risk groups had a lower TIDE score and a higher IPS score, implying that low-risk patients may be more sensitive to immunotherapy (35, 36). Next, to identify the efficacy of AAG_score as a biomarker to predict therapeutic response in GC patients, we estimated the IC50 values of 138 drugs in TCGA-STAD patients. We discovered that patients with low AAG_scores may positively react to ATRA, gefitinib, gemcitabine, obatoclax.Mesylate, paclitaxel, sorafenib, and bosutinib, while patients with high AAG_scores maybe respond better to docetaxel, shikonin, KU.55933, and multiple targeted therapy agents, including axitinib, dasatinib, erlotinib, imatinib, lapatinib, and nilotinib (**Figures 9F–L**). Overall, these findings indicated that AAGs were correlated with drug sensitivity.

## Discussion

Angiogenic cytokines are critical pro-angiogenesis drivers, as well as important immune regulators. Angiogenic cytokines can regulate angiogenic switches as activators or inhibitors during tumor progression in GC (37). And angiogenic cytokines secreted by GC cells activate endothelial cells and autocrine loops to modulate tumor development (38). Additionally, angiogenic cytokines contribute to immune suppression by inhibiting antigen-presenting cells and immune effector cells, or by activating suppressing immune cells (such as Treg and tumor-associated macrophages). These suppressive immune cells can in turn stimulate angiogenesis, resulting in a vicious pattern of impaired immune activation (39). Accumulative evidence has demonstrated the inevitable association between angiogenesis and intrinsic immunity, and angiogenesis targeting may serve a critical role in enhancing cancer immunotherapy (40, 41). However, numerous reports have only emphasized a single AAG or a specific immune cell subtype. Therefore, it is necessary to further clarify the holistic impact and TME infiltration features regulated by the combinatorial action of diverse AAGs.

In this research, we identified the transcriptional alterations and expression of AAGs on the basis of the TCGA–STAD cohort. Despite the low mutational intensity of AAGs, most of them are up-regulated in GC patients and associated with prognosis. We then divided GC patients into two angiogenesis subgroups (Cluster A and B) with the unsupervised clustering approach. There were obvious discrepancies in clinical outcomes, immune infiltrations, and functions between the two subgroups. Gene mutations in GC may serve a leading role in the response to immunotherapy. Based on the DEGs related to the subgroups signature, three gene clusters with different clinical features, immune activities, and functions were created for GC. By LASSO Cox regression, AAG_score was established to quantify the angiogenesis subgroups. The cluster A and gene cluster A with the poorest clinical outcomes had the greatest AAG_score among AAG_clusters and three gene clusters. Interestingly, patients with a high AAG_score had unfavorable OS, suggesting that a high AAG_score could predict an unfavorable prognosis. Angiogenesis is involved in the malignant behavior of diverse tumors, including GC (42, 43). Consistently, our GSEA findings demonstrated that cancer- and metastasis-associated pathways were markedly enriched, confirming the existing conclusions.

AAG_score was remarkedly relevant to clinicopathological features of GC. After controlling confounding parameters, the results indicated that AAG_score was an independent predictor for GC patients’ survival outcomes. ROCs validated its predictive robustness for 1-, 3-, and 5-year OS. Recently, an angiogenesis-associated risk score has been established for the clinical outcomes of GC patients. Accordingly, AAG_score may have a reliable predictive capacity for patients’ prognoses. The aggregation of gene mutations results in carcinogenesis, which is associated with neo-angiogenesis. Our results proved that there was a significant discrepancy in genomic alterations between low and high AAG_scores. Higher TMB has been validated to be related to a better prognosis for GC patients, consistent with our findings (44). The clinical outcomes in the low AAG_score group were evidently superior to those in the low TMB groups, suggesting AAG_score could be utilized to independently predict the responsiveness of immunotherapy.

Immune interactions are critical characteristics of tumorigenesis and therapeutic target for GC. Stromal cells and immune cells are the primary elements of the TME, and immune and stromal scores are related to clinic characteristics and prognosis in GC (45, 46). We calculated these scores with the ESTIMATE algorithm and found that a high AAG_score group obviously presented higher immune and stromal scores than a low AAG_score group. This suggested that angiogenesis could be associated with the involvement of the TME, thus regulating neoplastic occurrence and development. We identified that higher enrichment of T cells (T helper, CD 4+ and CD 8+T cells) and DCs were correlated with low AAG_score. The enrichment of Tregs, inhibiting the anti-tumor immunoreactivity, was related to poor survival (47). This is concordant with our findings of abundant Tregs in the TME of patients with high AAG_scores. Previous reports also demonstrated that angiogenesis factors may serve as immune modulators, and the immune system could participate in carcinogenesis by inducing pathological vascularization (48, 49). Therefore, targeting angiogenesis may be a valuable regulative strategy for immunotherapy of GC.

At present, GC is gradually resistant to chemotherapy (50). This study identified the potential sensitive drugs for patients in different AAG_socre groups, and the combination of these drugs and targeting angiogenesis may contribute to alleviating drug resistance and improving clinical outcomes. Furthermore, the effectiveness of immunotherapy requires specific biomarkers as a predictive pattern. TIDE and IPS signatures have been created to evaluate ICIs response. Accordingly, we observed that GC patients with low AAG_scores displayed low TIDE scores and positive responsiveness for anti-PD1 and anti-CTLA-4 therapy. Elevated levels of diverse immune cell infiltration were also found in low AAG_scores. This demonstrates that AAG_score has the potential to determine patients who have a better response for ICB.

This study has several limitations. Data from public databases are obtained retrospectively, and inherent selection bias may affect their robustness. And additional clinical variables should be introduced into the study to fully explore the clinical value of AAG_scores. Furthermore, extensive prospective studies and complementary *in vivo* and *in vitro* experimental studies are necessary to gain insight into the relationship between risk scores and TME, thus confirming our findings.

## Conclusion

Briefly, our systematic analysis of AAGs demonstrates a comprehensive regulatory strategy, and thus influences TME, prognosis, and clinical characteristics of GC patients. We also clarify the potency of AAGs as a biomarker of therapeutic response. Our study reveals the critical clinical significance of AAGs and offers a valuable basis for further researches on personalized therapy in GC patients.

## Data Availability Statement

The datasets presented in this study can be found in online repositories. The names of the repository/repositories and accession number(s) can be found in the article/**Supplementary Material**.

## Author Contributions

All authors contributed to the study’s conception and design. XQ, WX, and SL performed data collection and analysis. XQ and WX wrote the manuscript. YZ polished and revised the manuscript. All authors commented on previous versions of the manuscript and read and approved the final manuscript.

## Funding

This study was supported by the National Natural Science Foundation of China (No. 81872255, 62041101), Jiangsu Provincial Maternal and child health scientific research project (No. F202005) and the Key Medical Talents Foundation of Jiangsu Province (No. 2016KJQWZDRC-03).

## Conflict of Interest

The authors declare that the research was conducted in the absence of any commercial or financial relationships that could be construed as a potential conflict of interest.

## Publisher’s Note

All claims expressed in this article are solely those of the authors and do not necessarily represent those of their affiliated organizations, or those of the publisher, the editors and the reviewers. Any product that may be evaluated in this article, or claim that may be made by its manufacturer, is not guaranteed or endorsed by the publisher.
