# Supplementary material for: Molecular Characteristics, Clinical Significance, and Cancer Immune Interactions of Angiogenesis-Associated Genes in Gastric Cancer
Source: Front Immunol. 2022 Feb 22;13:843077. doi: 10.3389/fimmu.2022.843077 (PMC8901990; doi:10.3389/fimmu.2022.843077)
Supplement: Supplementary file 1 [file DataSheet_1.zip › Supplementary_Figures.docx]

**Supplementary Figures**


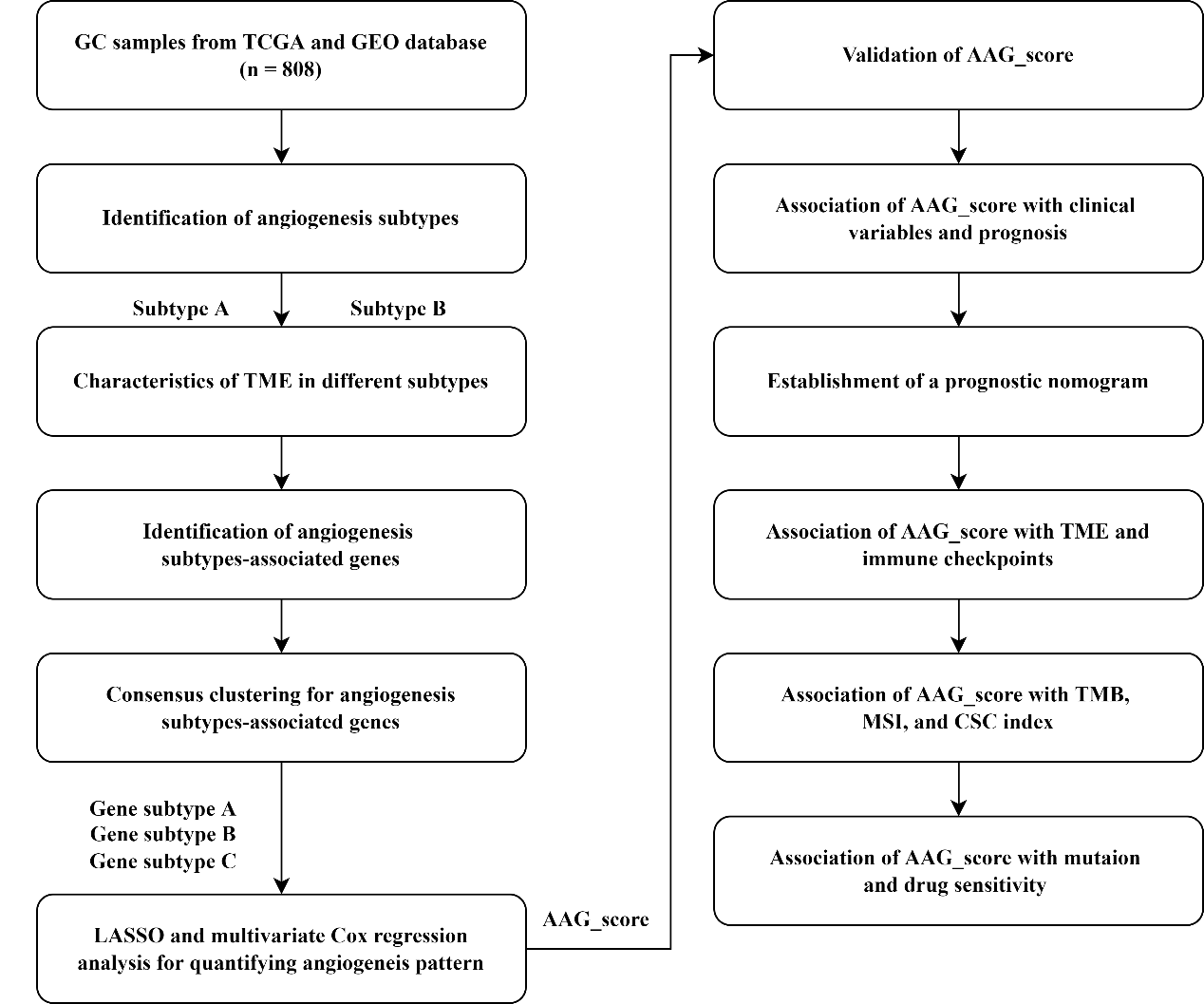


**Figure S1** The entire analytical process of the study.


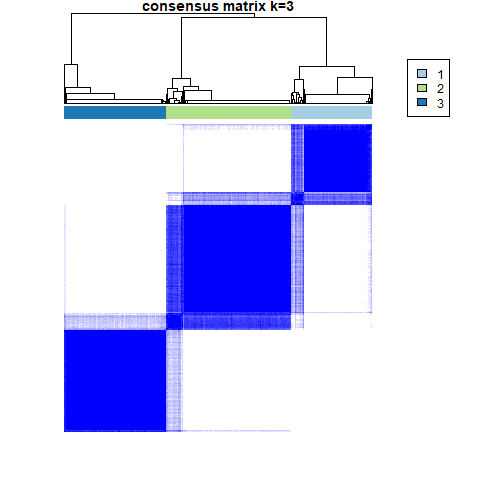


**Figure S2** Identification of gene subtypes based on DEGs among two angiogenesis subgroups in GC cohort.


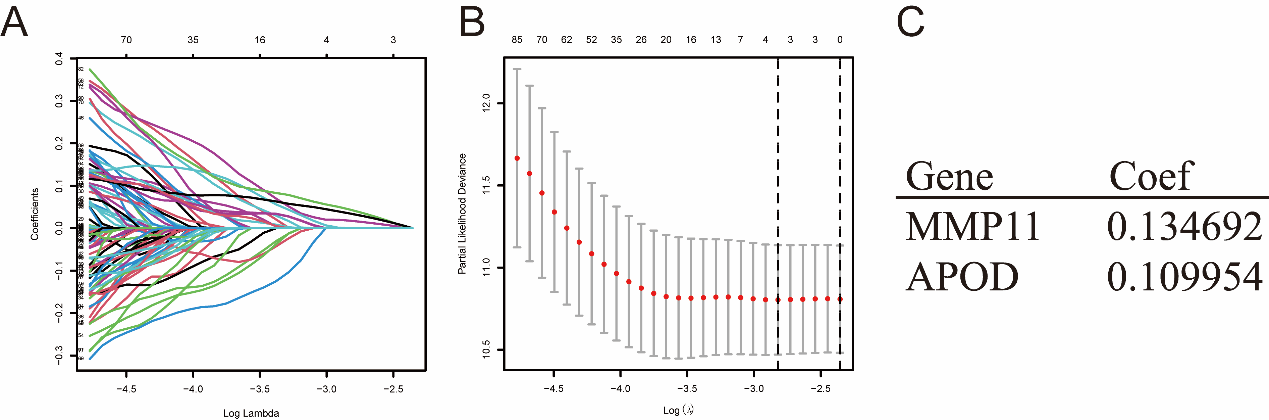


**Figure S3** Identifying representative candidate prognostic genes. (A-B) The LASSO regression analysis and partial likelihood deviance on the prognostic genes. (C) The results of multivariate cox regression analysis for prognostic genes.


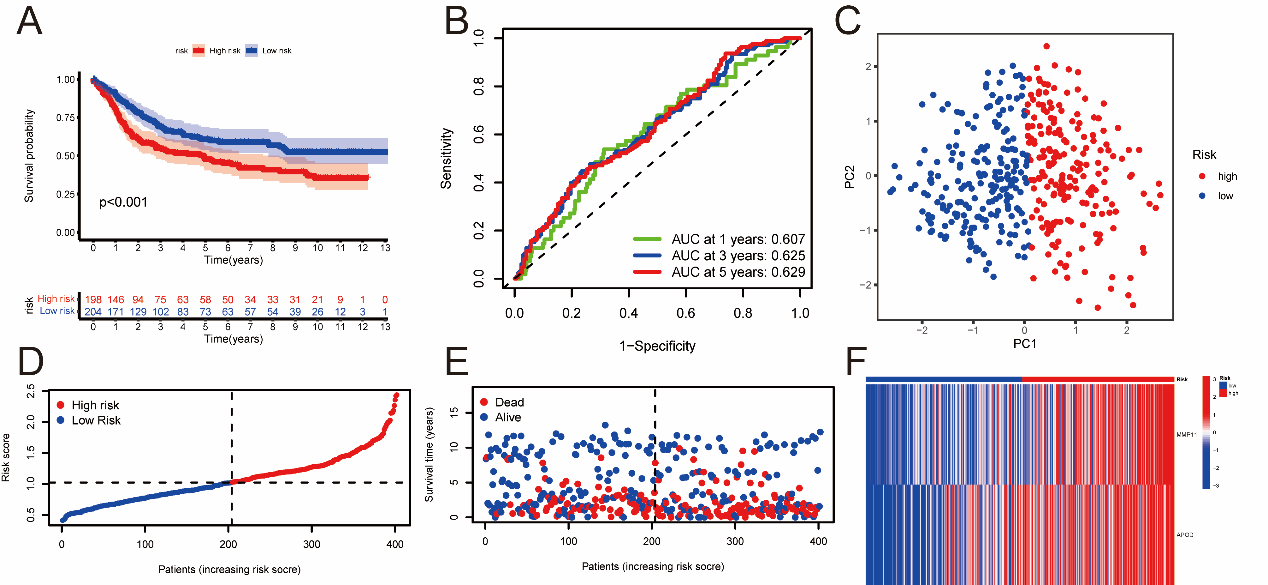


**Figure S4** Validation of AAG_score in test cohort. (A) KM analysis of the OS between the two groups. (B) ROC curves to predict the sensitivity and specificity of 1-, 3-, and 5-year survival according to the AAG_score. (C) The PCA analysis demonstrated that the patients in the different risk groups were distributed in two directions. (D-E) The ranked dot plot indicates the AAG_score distribution and scatter plot presenting the patients’ survival status. (F) Expression patterns of 2 selected prognostic genes in high- and low-risk groups.


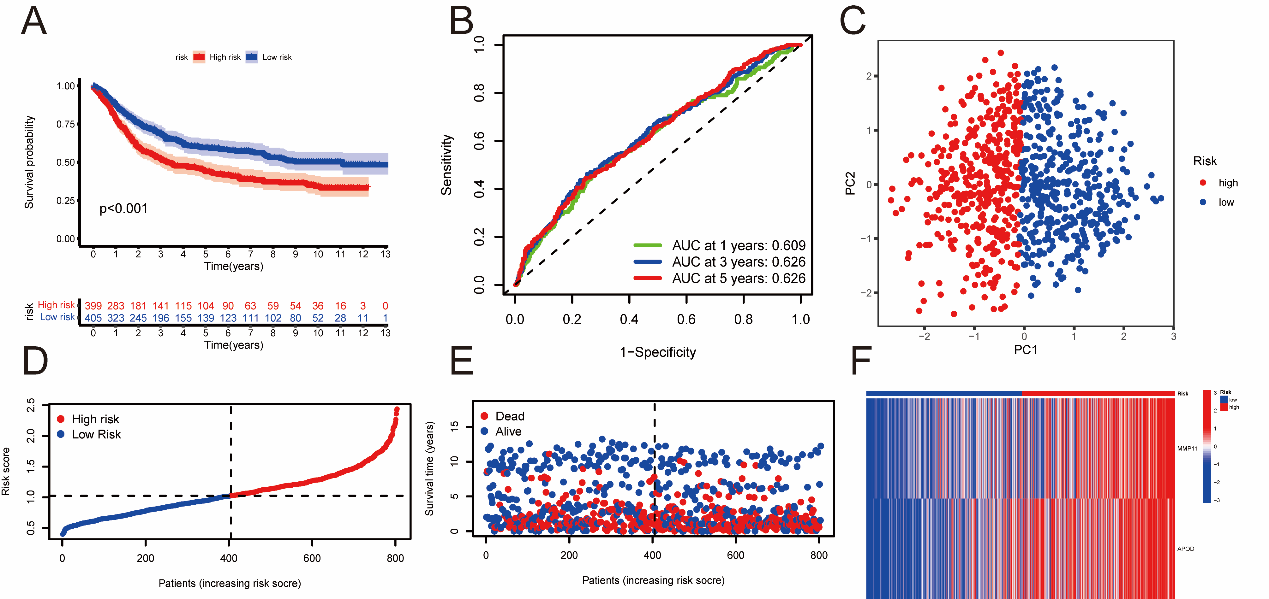


**Figure S5** Validation of AAG_score in entire cohort. (A) KM analysis of the OS between the two groups. (B) ROC curves to predict the sensitivity and specificity of 1-, 3-, and 5-year survival according to the AAG_score. (C) The PCA analysis demonstrated that the patients in the different risk groups were distributed in two directions. (D-E) The ranked dot plot indicates the AAG_score distribution and scatter plot presenting the patients’ survival status. (F) Expression patterns of 2 selected prognostic genes in high- and low-risk groups.


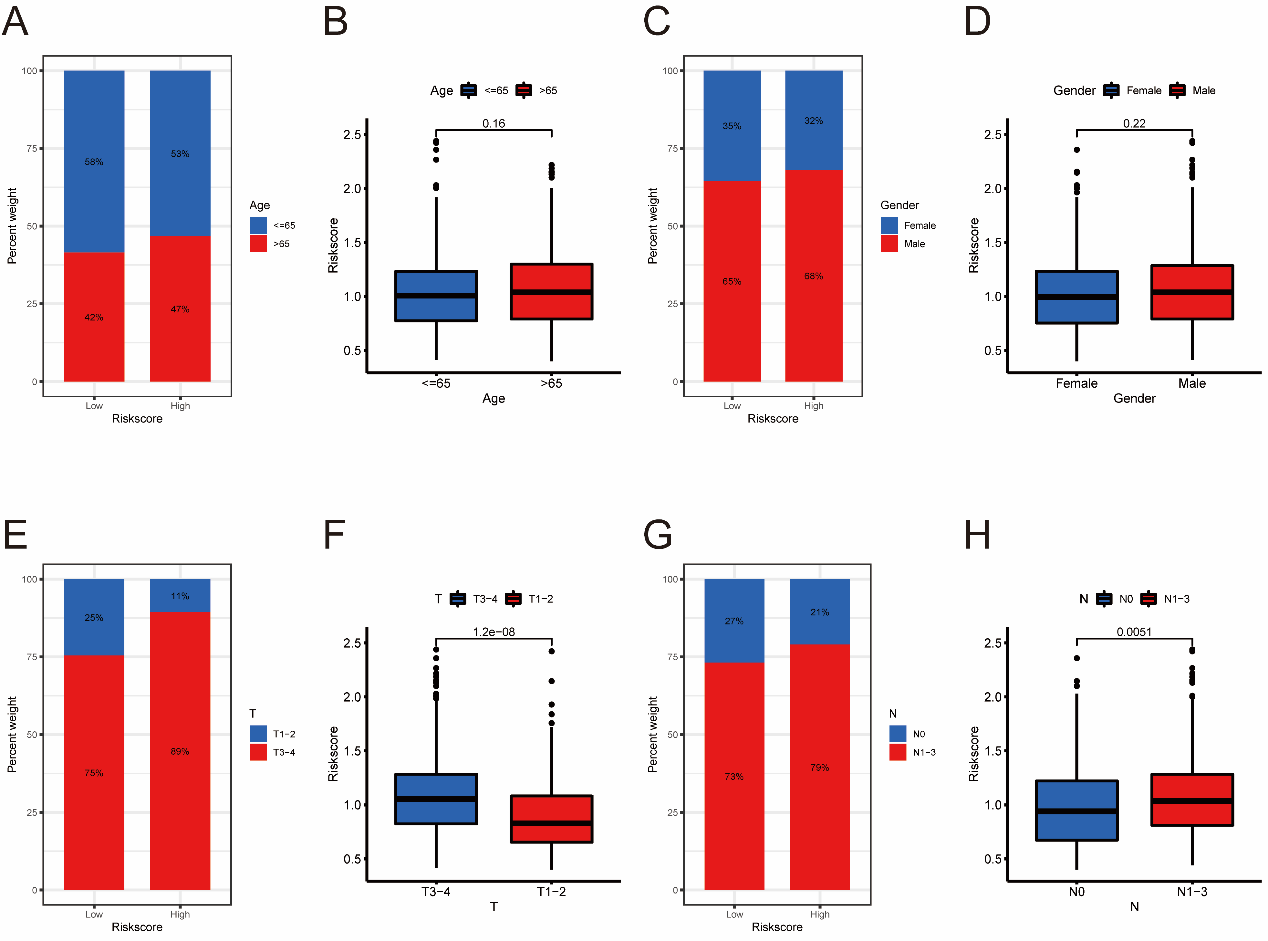


**Figure S6** The correlation analysis of AAG_score and clinicopathological variables in GC. The (A) Age, (C) Gender, (E) T-stage, and (G) N-stage distribution of with patients in different risk groups. The correlation between the AAG_score and (B) Age, (D) Gender, (F) T-stage, and (H) N-stage.


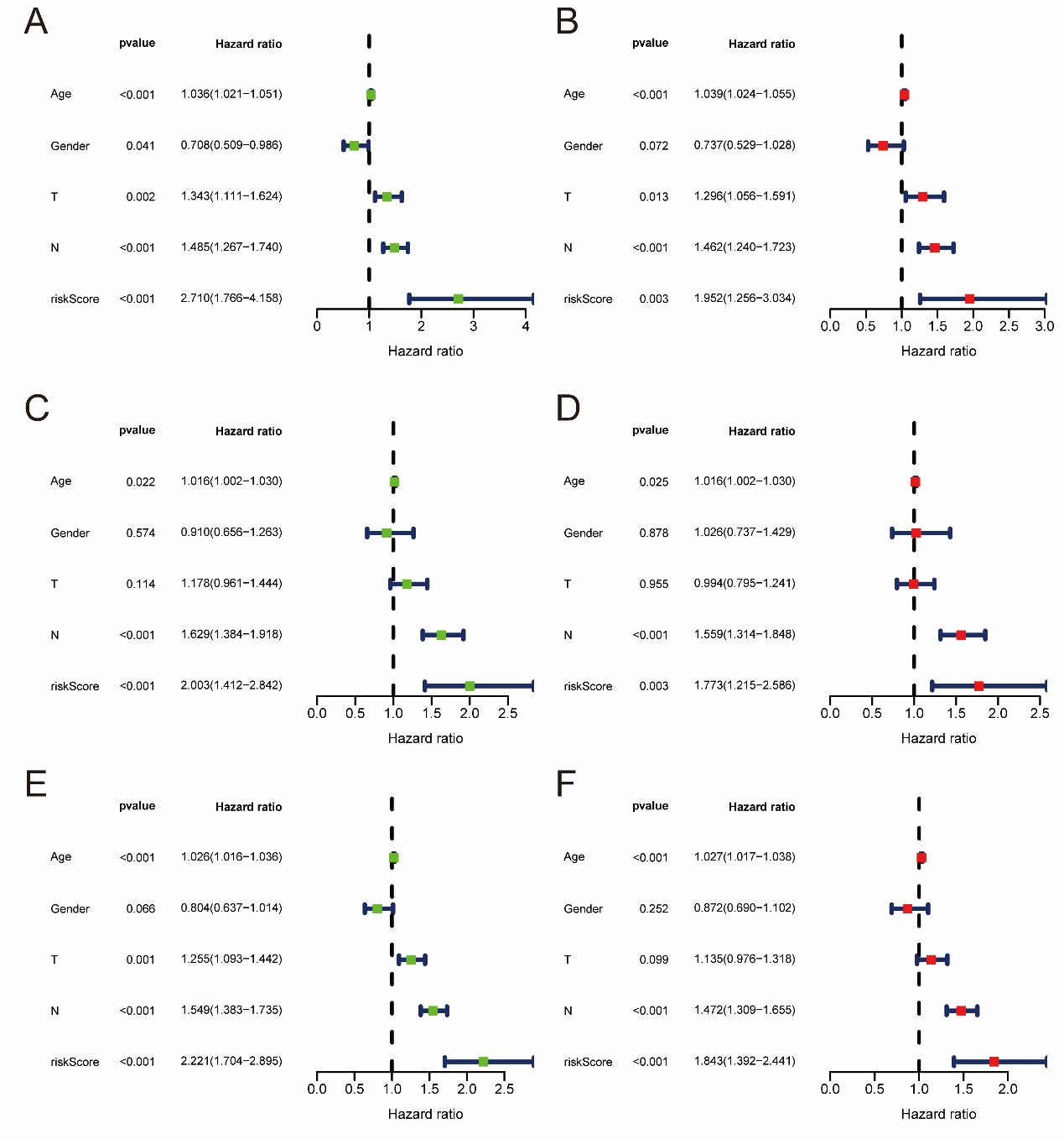


**Figure S7** The independent prognosis analysis of AAG_score and clinicopathological variables in GC. (A-B) Univariate and multivariate analyses showed the prognostic value of the AAG_score in the training cohort. (C-D) Univariate and multivariate analyses showed the prognostic value of the AAG_score in the test cohort. (E-F) Univariate and multivariate analyses showed the prognostic value of the AAG_score in the entire cohort.


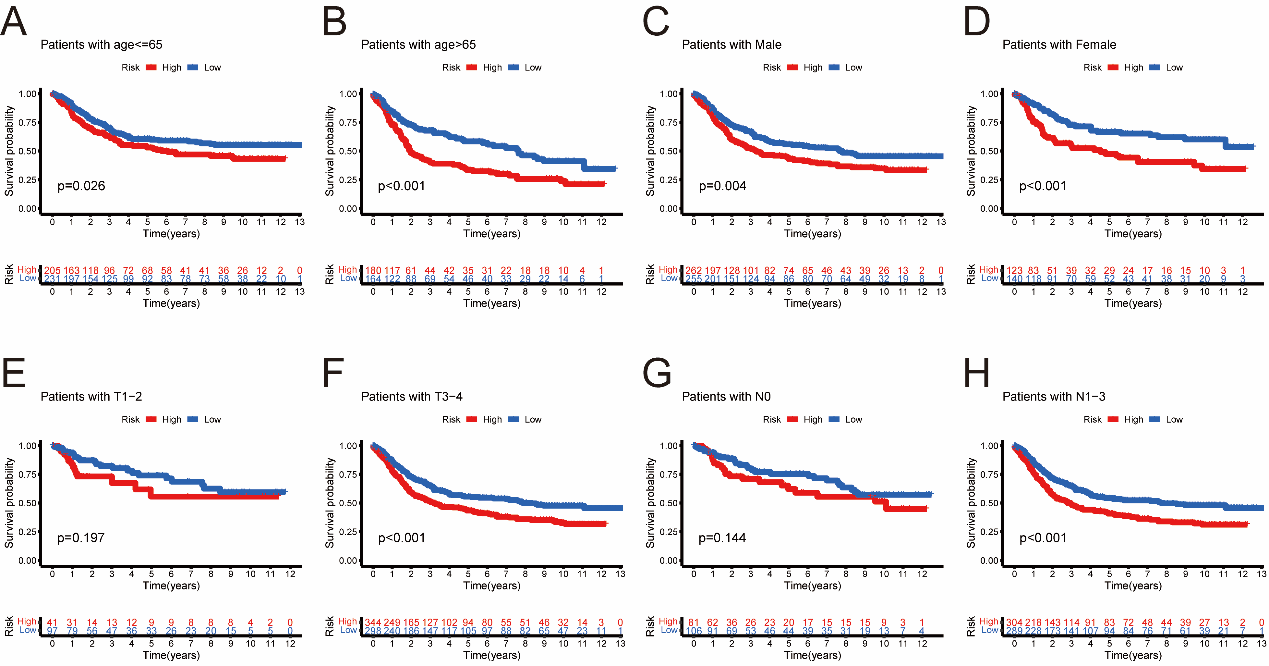


**Figure S8** Stratification analysis of the AAG_score in GC. (A-B) Age (age ≤ 65 and age > 60 years old). (C-D) Gender (male and female). (E-F) T-stage (T1-2 and T3-4). (G-H) N-stage (N0 and N1-3).


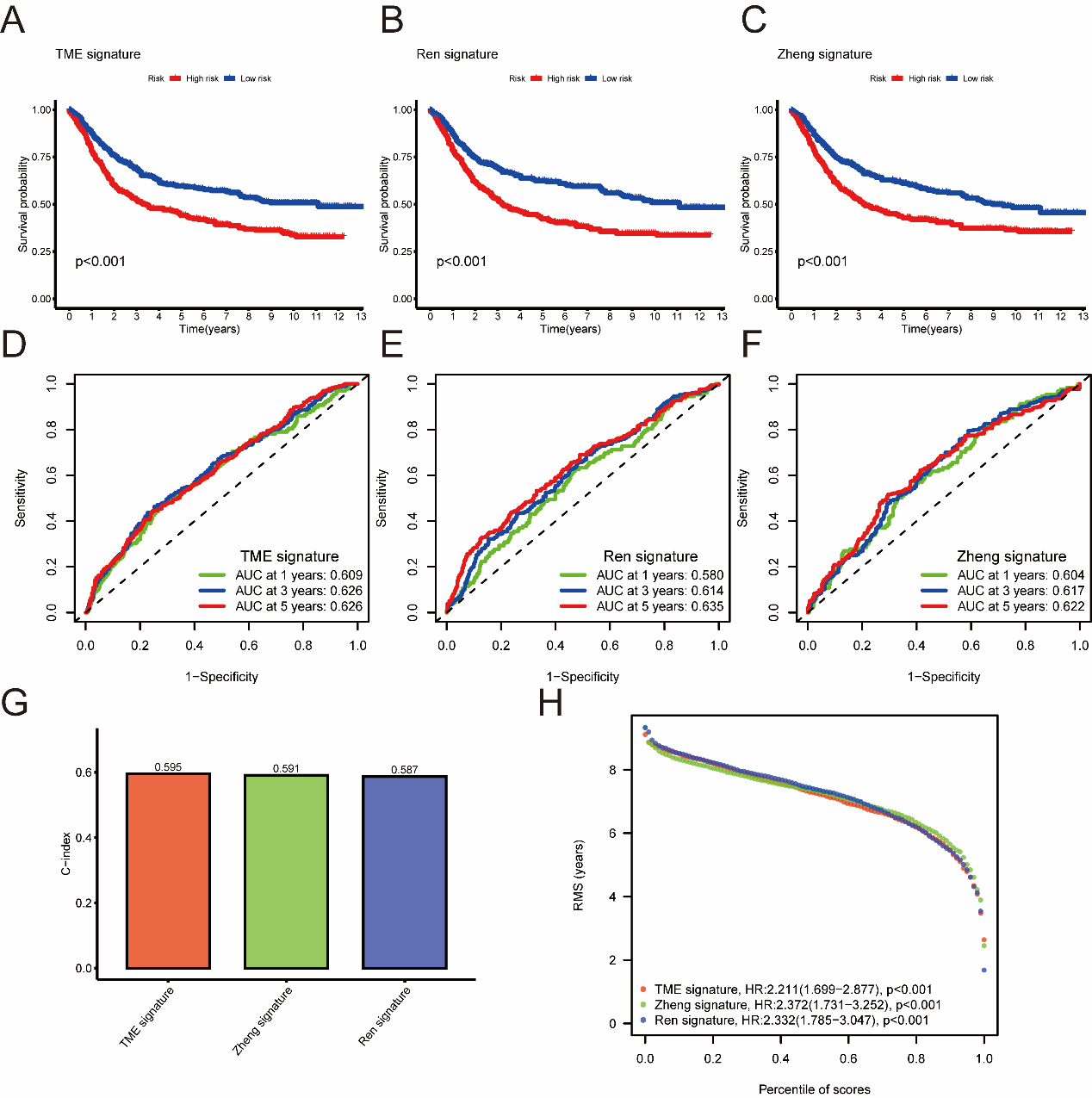


**Figure S9** Comparison of the signature in this study with prognostic signatures previously reported. (A, C) Kaplan-Meier survival analysis and ROC curves of TME signature. (B, D) Kaplan-Meier survival analysis and ROC curves of Ren signature. (C, F) Kaplan-Meier survival analysis and ROC curves of Zheng signature. (G, H) C-index and RMS of the three prognostic signatures.

**
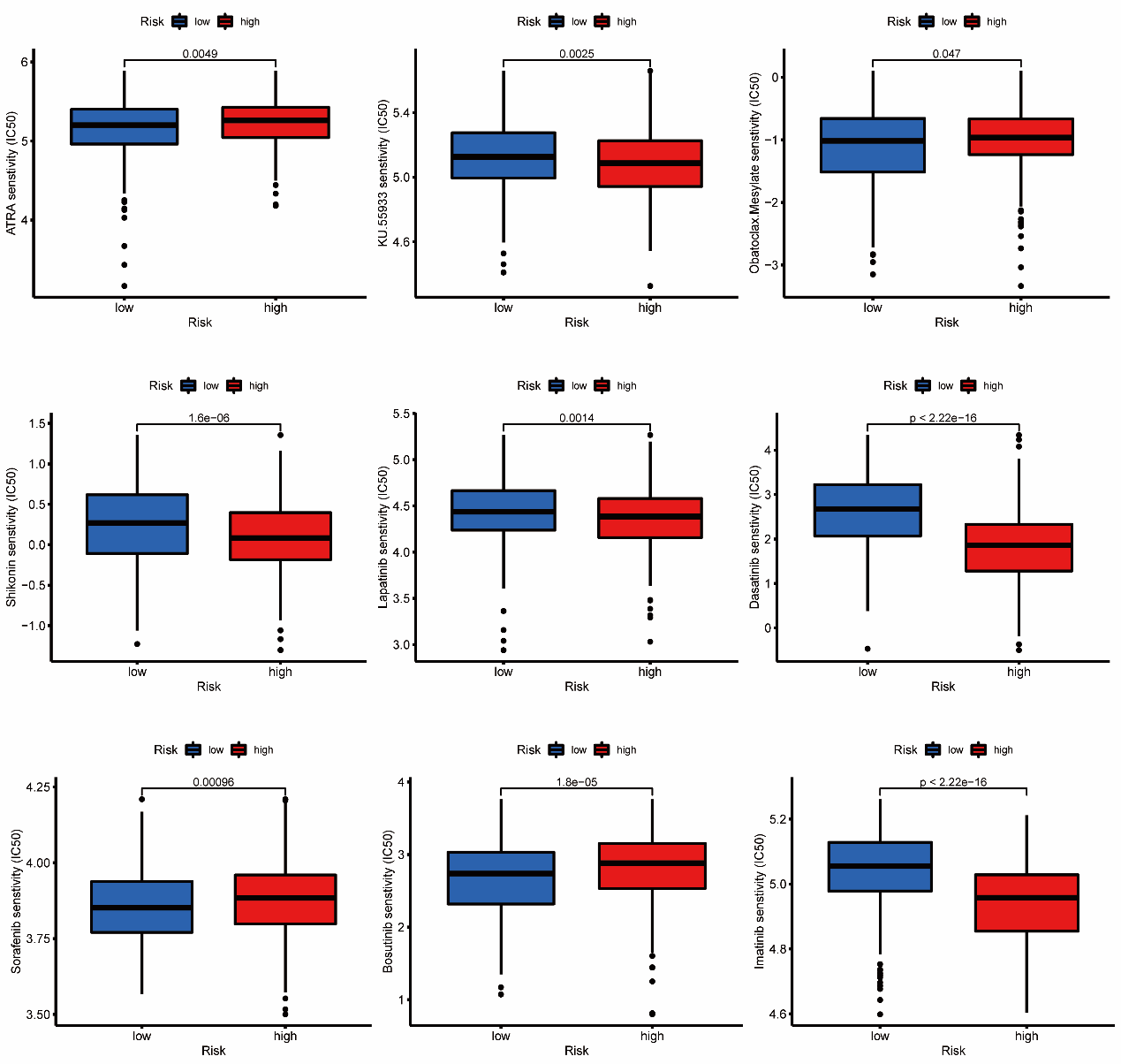
**

**Figure S10** Drug sensitivity analysis of common chemotherapeutic drugs.
